# Histone H3 Post-Translational Modification Changes are Linked to Manganese and Copper Exposure in *Saccharomyces cerevisiae*

**DOI:** 10.17912/micropub.biology.002104

**Published:** 2026-05-21

**Authors:** William Villasi, Rania Frederic, Sidra Qureshi, Boyan Zheng, Mariana P. Torrente, Raven M.A. Fisher

**Affiliations:** 1 Department of Chemistry and Biochemistry, Brooklyn College, Brooklyn, NY, US; 2 Ph.D. Programs in Biology, Biochemistry, and Chemistry, CUNY Graduate Center, New York, NY, US; 3 Ph.D. Program in Biochemistry, CUNY Graduate Center, New York, NY, US

## Abstract

Prolonged exposures to heavy metals are risk factors for chronic diseases, such as Amyotrophic Lateral Sclerosis and Frontotemporal dementia (ALS/FTD). ALS/FTD comprises a fatal neurodegenerative disease continuum and is linked to disruptions in the levels of histone post-translational modifications (PTMs). Epigenetic mechanisms can connect environmental exposures to disease occurrences. Here, we examine the effects of manganese and copper exposure on the H3 PTM landscape in yeast. Manganese exposure decreases H3K9ac, H3K14ac, and H3S10ph levels. Copper exposure increases H3S10ph and H3K14ac levels and decreases H3K36me3 levels. This provides a basis for linking environmental exposure to biological mechanisms of disease.

**Figure 1. Manganese and Copper Exposure Connect to Alterations in Distinct Histone H3 PTMs in yeast:** Column bar graphs and representative western blots for A) H3K9ac, C) H3K14ac, E) H3S10ph, and G) H3K36me3 after manganese treatment, n = 3-4. Column bar graphs and representative western blots for B) H3K9ac D) H3K14ac, F) H3S10ph, and H) H3K36me3 levels after copper treatment, n = 3-7, * = p < 0.05 ** = p < 0.01 *** = p < 0.001. I) Manganese and Copper exposure lead to distinct H3 PTM profiles. Figure panel created with Biorender.com

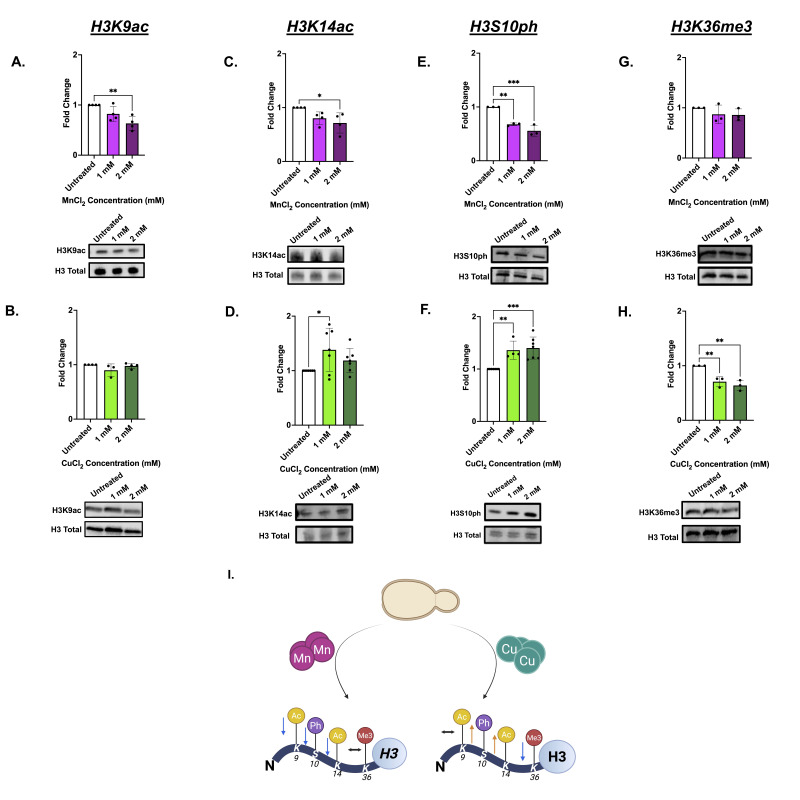



## Description

Exposure to certain environmental toxins, including various heavy metals, has been linked to permanent organ damage, cancer, as well as neurodegenerative diseases like Amyotrophic Lateral Sclerosis (ALS) and Frontotemporal dementia (FTD) (Jaishankar et al., 2014; Gorini and Tonacci, 2024; M. Wang et al., 2017). Amyotrophic Lateral Sclerosis (ALS) is a fatal and incurable neurodegenerative disease that leads to the loss of motor neurons (Brown and Al-Chalabi, 2017). Frontotemporal dementia (FTD) is an Alzheimer’s Disease-related dementia that causes the degeneration of the frontal and temporal lobes of the brain and is the leading cause of dementia in people under 65 years of age (Bang et al., 2015). Due to pathological and genetic overlap, ALS and FTD are recognized to form a disease continuum (Burrell et al., 2016). Most cases of ALS and FTD occur sporadically, but a small percentage of patients have a family history of disease linked to mutations in several genes, such as Fused in Sarcoma (FUS), TAR DNA-binding protein 43 *(* TDP-43), Cu-Zn Superoxide Dismutase 1 (SOD1), and Chromosome 9 open reading frame 72 (C9orf72)(Abramzon et al., 2020). These mutations form different proteinopathies, or aggregations of proteins that can propagate diseases after the three-dimensional conformational change and self-association of normal proteins (Bayer, 2013). Both familial and sporadic cases are linked to the misfolding and aggregation of proteins that lead to neurodegeneration (J. Wang et al., 2025). However, exact disease mechanisms and etiology remain unknown (Ghasemi and Brown, 2018).

Uncovering the role of heavy metals in disease is of particular relevance due to frequent environmental and occupational exposure (Santurtún et al., 2025). Two examples of heavy metals include manganese and copper, which are essential for cellular processes but become toxic at high levels and/or prolonged exposure (Fisher and Gupta, 2024). High levels of copper and manganese are associated with increased prevalence of ALS/FTD, but it is not yet known if this association is causative (Santurtún et al., 2025; Jang et al., 2024). Previous studies have linked heavy metal exposure with both familial and sporadic ALS/FTD due to their genotoxic effects and propagation of mutations (Kim et al., 2025). Additionally, the accumulation of toxic metals is thought to lead to neuronal and astrocytic dysfunction, but exact biological processes underlying how metal exposure may connect to disease pathology are still unknown (Gorini and Tonacci, 2024). 

ALS/FTD has been previously connected to perturbations in various epigenetic mechanisms (Belzil et al., 2017; Bennett et al., 2019b; van Zundert and Montecino, 2025). Epigenetics refers to heritable changes to phenotypes that do not arise from changes to DNA (Allis and Jenuwein, 2016). Epigenetic mechanisms such as DNA methylation and histone post translational modifications (PTMs) alter gene expression and phenotype by altering the structure and composition of chromatin (Allis and Jenuwein, 2016). Chromatin consists of a histone octamer core wrapped by DNA with a protruding N-terminal tail that undergoes addition of chemical groups such as methylation, acetylation, and phosphorylation. Chromatin can be roughly divided into two states: heterochromatin, in which the DNA is tightly wound around the histone and is transcriptionally silent, and euchromatin, where the DNA is loosely wrapped around the octamer and is transcriptionally active. Histone PTMs can tighten or loosen the DNA wound around the histones and act as binding platforms for other proteins. These PTMs are site-specific and can affect the transcriptional activity of specific genes (Bannister and Kouzarides, 2011).

Different ALS/FTD proteinopathies are associated with unique histone PTMs changes. In FUS ALS/FTD yeast models, there is a decrease in the levels of transcriptionally active PTMs, H3S10ph and H3K14ac (Bennett et al., 2021). On the other hand, in TDP-43 yeast models, there are increases in H4K12ac and H4K16ac levels with decreases in the levels of H3K36me3 (Chen et al., 2018). These changes may be linked to genomic instability and heterochromatin breakdown (Fisher and Torrente, 2024). Moreover, SH-SY5Y cells overexpressing SOD1 display decreased levels of H3S10ph-H3K14ac and H3K4me2, suggesting that SOD1 mutations lead to increased formation of heterochromatin (Masala et al., 2018). Therefore, ALS/FTD may affect transcriptional activity through histone PTMs.

Here, we explore the impact of heavy metals on histone PTMs. Exploiting yeast as a model system, we seek to uncover how manganese and copper exposure impact histone PTMs to further understand the biological conduits linking heavy metal exposure to ALS/FTD development. Yeast, although lacking a nervous system, can be easily manipulated and has highly conserved cellular processes, making it ideal for uncovering mechanisms associated with neurodegeneration (Pereira et al., 2012; Cobos et al., 2025).

In W303 yeast treated with 1 and 2mM of manganese (Mn) and copper (Cu), we exploited western blotting to characterize changes of Histone H3 PTM levels. These concentrations were used to simulate pathological concentrations and chronic exposure to these metals, as heavy metals can be stored long-term in different tissues, such as the liver, bone tissue, and fingernails, which can potentially propagate neurodegeneration (Gaetke et al., 2014;Rolle-McFarland et al., 2020). After overnight pre-treatment, we cultured Mn and Cu-treated yeast for 5 hours with continued treatment to achieve mid-log phase (Salari and Salari, 2017). We focused on modifications on Histone H3 as it is the most pervasively modified. Furthermore, we selected H3 modifications associated with gene regulation and chromatin state (Grant, 2001).

In Mn-treated yeast, we found moderate decreases in the genome-wide levels of H3K9ac, H3K14ac, and H3S10ph ( **Figures 1A, 1C, and 1E** ). Interestingly, manganese treatment did not affect other gene-regulatory PTMs, such as H3K27ac and H3K79me3, highlighting a site-specific role for metal-induced epigenetic dysregulation ( **Extended Data 1A and 1C** ). Furthermore, Mn treatment did not affect H3K36me3 levels ( **Figure 1G** ). While genome-wide PTM analyses are robust, differences at specific loci are likely to be much more pronounced due to the gene-specific roles of most PTMs (van Leeuwen and van Steensel, 2005). Like H3S10ph and H3K14ac, H3K9ac is an active promoter mark and is often found on highly expressed genes (Filleton et al, 2015). Therefore, decreases in H3K9ac and H3K14ac, as well as H3S10ph, suggest that manganese exposure may decrease transcriptional activity of genes (Filleton et al, 2015). This finding agrees with previous studies linking Mn exposure to hypoacetylation of H3 facilitated by increased histone deacetylase (HDAC) activity and decreased histone acetyltransferase activity (HAT) (Guo et al, 2018). Furthermore, Mn treatment of yeast resulted in the reduction of mRNA and rRNA levels, preventing translation (Hernandez et al, 2019). Overall, these findings support that Mn-induced hypoacetylation inhibits transcription and translation of genes, which may play a role in neurotoxicity and neurodegeneration (Hernandez et al, 2019).

In contrast, in Cu-treated yeast, we find about 40% increases in H3K14ac and H3S10ph levels ( **Figures 1D and 1F** ). However, we find no significant changes in H3K9ac following copper treatment ( **Figure 1B** ). H3K27ac and H3K79me3 also remain unchanged upon copper exposure ( **Extended Data 1B and 1D** ). H3K14ac is linked to active gene expression at promoters, suggesting that copper induces increased gene transcription (Regadas et al., 2021). Indeed, H3S10ph and H3K14ac typically co-occur and are seen as dual markers for gene activation (Winter et al., 2007). Although H3S10ph is an active mitosis mark involved in chromosomal condensation, both H3K14ac and H3S10ph were found to induce euchromatin formation during interphase, allowing for easier transcription (Komar and Juszczynski, 2020). This suggests that copper induces the transcription of genes through the promotion of euchromatin and reduction of heterochromatin formation (Kreuz and Fishele, 2016). These findings mirror previous studies that show that heavy metal stress induces increased nucleic acid and protein synthesis in yeast as a compensatory mechanism to resist copper stress (Kan et al., 2019). Excess copper ions produce reactive oxygen species (ROS), which necessitate the production of chaperones and copper-mediating proteins like SOD1 and CUP1, to prevent cellular damage (Shen et al., 2001; Kan et al., 2019).

Interestingly, unlike manganese exposure, we find that copper exposure is connected to decreases in the levels of H3K36me3 ( **Figure 1H** ). H3K36me3 is associated with DNA repair, as it occurs upon DNA damage and facilitates double-strand break repair through homologous recombination (Sharda and Humphrey, 2023). A decrease in H3K36me3 levels suggests dysregulation of DNA repair mechanisms (Sun et al., 2020). DNA repair interference permits the proliferation of DNA damage caused by oxidative stress and promotes the development of neurodegenerative diseases such as ALS (Kok et al., 2021). Hence, we hypothesize that prolonged copper exposure both promotes DNA damage and inhibits DNA repair pathways through ROS formation and epigenetic mechanisms.

Altogether, our findings suggest that manganese and copper exposures have distinct effects on the genome-wide levels of H3 modifications associated with gene transcription, chromatin structure, and DNA repair ( **Figure 1I** ). Effects on the histone PTM landscape due to heavy metal exposure can provide a biological mechanism underlying environmentally induced sporadic and familial neurodegeneration (Kulcsárová et al., 2025). While histone PTMs are understudied in the context of sporadic ALS, patient peripheral blood mononuclear cells (PBMCs) have increased chromatin accessibility (Kühlwein et al., 2023). Increased chromatin accessibility is typically linked to global histone hyperacetylation, which is consistent with copper exposure in yeast. Furthermore, in familial ALS SOD1 transgenic mice, there was a decrease in H3S10ph-H3K14ac levels, which is seen in Mn-treated yeast (Masala et al., 2018). Additionally, FUS overexpression in yeast is connected to decreases in H3S10ph, H3K14ac, and H3K56ac levels, which are also in line with manganese exposure in yeast (Chen et al., 2018).

H3 changes associated with copper exposure were also in accordance with H3 alterations connected to C9orf72 dipeptide repeat proteinopathy. In (PR) _50,_ overexpression yeast there was also an increase in H3S10ph and H3K14ac levels (Cobos et al., 2025). We postulate that exposure to manganese and copper can worsen histone PTMs changes linked to ALS/FTD proteinopathies and lead to disease progression through epigenetic mechanisms. Follow-up studies elucidating the compounding effects of treating yeast ALS/FTD proteinopathy models with manganese and copper could explore the interplay between genetic and environmental factors in disease progression. Additionally, studies exploring histone PTMs and the effects of heavy metals on the epigenome in sporadic models are needed. While our work is currently limited to yeast models and must be verified in mammalian models, it reveals a potential biological avenue for environmental exposures to increase propensity for neurodegenerative disease and provides openings for disease diagnostics, prevention, and pharmacological intervention.

## Methods

**Yeast Culture**

All yeast strains were W303 yeast ( *MATa, can1-100,his3-11,15,leu2,3,11,12,trp1-1,ura3-1,ade2-1)* (Sanchez and Lindquist, 1990). Prior to all experiments, yeast was streaked onto YPD and incubated at 30 °C for 2–3 days. Yeast was grown overnight in YPD supplemented with 1 and 2mM MnCl _2_ or CuCl _2_ at 30 °C at 150 rpm. Yeast grown in YPD without metals (untreated) was used as a control. Liquid yeast cultures were standardized to an OD _600_ of 0.3 and grown in YPD supplemented with MnCl _2_ or CuCl _2_ for an additional 5 h at 30 °C at 150 rpm until an OD _600_ of 0.6–0.8 (Cobos et al., 2025). The cells were pelleted, flash-frozen using liquid nitrogen, and stored in –80° C for subsequent use (Bennett et al., 2019a).

**Western Blot**

Frozen cell pellets were prepared for western blotting as previously described (Cobos et al., 2025). Pellets were lysed with 0.2 M NaOH and β-mercaptoethanol on ice for 10 minutes. Cells were then resuspended in 100 mL of 1X SDS sample buffer. The cell suspensions were boiled for 10 minutes at 95 °C and then resolved in a 15% polyacrylamide gel. Gels were transferred onto PVDF membrane (EMD Millipore, Taunton, MA) and subsequently incubated for one hour at room temperature with BSA blocking buffer (LICOR Biosciences, Lincoln, NE). The PVDF membranes were then incubated overnight at 4 °C with primary antibodies, H3K9ac 1:500 (Abcam, cat no. ab10812), H3K14ac 1:5000 (Millipore, cat no. ab 07-353), H3K27ac 1:1000 (Abcam, cat no. ab177178), H3S10ph 1:1000 (Abcam, cat no. ab5176), H3K36me3 1:1000 (Abcam, cat no. ab9050), H3K79me3 1:1000 (Abcam, cat no. ab2621), and H3 total 1:2000 (Abcam, cat no. ab24834).

After incubation of the membrane in primary antibody, the membrane was washed with 1X TBST five times for five minutes before incubating in secondary antibodies: donkey anti-mouse (1:10,000; LICOR Biosciences, cat no. 926-32212) and donkey anti-rabbit (1:20,000; LICOR Biosciences, cat no.926-68073) for one hour at room temperature. The membrane was then washed with 1X TBST four times for five minutes and 1X TBS one time for at least five minutes. All Western Blots were imaged on a LICOR Odyssey FC (LICOR Biosciences, Lincoln, NE). All trials were repeated a minimum of three times with completely independent cell samples (Bennett et al., 2019a).

**Statistical Analysis**

Densitometric analysis of Western blots was performed using Image Studio (LICOR Biosciences, Lincoln, NE). H3 total signals were used to standardize loading across all sets. The relative density of the histone modification signal was calculated by dividing the signal of a particular histone modification by the H3 total signal. The fold change of relative density of each metal concentration versus the untreated control was calculated by dividing the relative density of each histone modification at each concentration by that of the untreated sample. These fold changes were then tested for statistical analysis. All statistical analysis was performed on GraphPad Prism (GraphPad Software, Boston, MA). Statistical analysis of the difference between the fold-change of the relative densities of each concentration to the untreated sample was performed using a one-way ANOVA test using p=0.05 as the cutoff for significance (Bennett et al., 2019a). Error bars represent the standard deviation (SD). 

## Reagents

All chemicals are from Sigma-Aldrich (St. Louis, MO, USA) unless otherwise specified.

## Data Availability

Description: Manganese and Copper Exposure do not Impact H3K27ac andH3K79me3 Levels.. Resource Type: Dataset. DOI: https://doi.org/10.22002/cg1gg-t4328
